# Walking Ability After Microsurgical Reconstruction of Pediatric Popliteal Pterygium Syndrome—A Case Report

**DOI:** 10.3390/jpm14111097

**Published:** 2024-11-07

**Authors:** Martin Aman, Mirjam Thielen, Ulrich Kneser, Leila Harhaus

**Affiliations:** 1 Department of Hand Surgery, Peripheral Nerve Surgery and Rehabilitation, Clinic of Plastic and Reconstructive Surgery, Burn Center, BG Trauma Center Ludwigshafen, Department of Hand- and Plastic Surgery, University of Heidelberg, 69117 Heidelberg, Germany; 2 Clinic for Hand, Replantation, and Microsurgery BG Klinikum Unfallkrankenhaus Berlin and Chair of Hand, Replantation, and Microsurgery at the Charité University Medicine Berlin, 10117 Berlin, Germany

**Keywords:** popliteal pterygium syndrome, knee flexion, pediatrics, congenital, microsurgery, nerve graft

## Abstract

Background: Popliteal pterygium syndrome (PPS) is a rare congenital disorder characterized by orofacial, cutaneous, musculoskeletal, and genital anomalies. Surgical interventions are necessary to address the severe knee flexion contracture and equinovarus deformity, but there are no established treatment guidelines. Methods: We present the case of a one-year-old patient with PPS and discuss the challenges in managing the knee deformity. The surgical option chosen for the unilateral knee contracture of 80° consisted of skin management by a large Z-plasty, lengthening of popliteal vessels by grafts, lengthening of the tibial and peroneal nerves by autografts and allografts, capsular releases, and tendon releases to improve mobility and preserve foot sensibility. Results: With a three-year follow-up, the surgical interventions resulted in proper ability to walk freely. Wearing of a foot orthesis was necessary to balance the leg length differences and support the midfoot deformity. Furthermore, sensation of the foot could be restored in terms of touch sensibility and perfusion was always stable during growth. Discussion: The treatment of PPS requires a multidisciplinary approach, considering the rarity and complexity of the syndrome. Surgical interventions aim to release contractures, correct deformities, and preserve foot sensibility. Each treatment option has its advantages and disadvantages, highlighting the need for individualized care.

## 1. Introduction

Popliteal pterygium syndrome (PPS) is an uncommon autosomal dominant congenital disorder characterized by a combination of orofacial, cutaneous, musculoskeletal, and genital anomalies. First described by Trelat in 1869 [1], PPS is diagnosed when at least three of the following conditions are present: cleft lip/palate, popliteal pterygium, paramedian lower-lip pits/sinuses, and genital and toenail abnormalities [2].

One of the hallmark features of PPS is a severe knee flexion contracture (PP—popliteal pterygium) accompanied by an equinovarus deformity. The popliteal pterygium, composed of a fibrous cord covered by a web of skin, typically extends from the ischial tuberosity to the heel. In some cases, the hamstrings and calf muscles may have unusual insertions along the skin web or may even be absent. The neurovascular bundle, comprising the popliteal vessels, sciatic nerve, and peroneal nerve, may either lie attached anteriorly to the cord in a shortened position or remain in its normal position in the popliteal fossa [3].

As a congenital malformation, PP can significantly impact an individual’s ability to ambulate, depending on the severity of the condition. Therefore, the management and treatment of PP pose significant challenges for surgeons. The primary objectives of treatment are to release the knee flexion contracture, correct the equinovarus deformity, and ensure foot sensibility is preserved to enable ambulation. However, due to the rarity of PPS, there are currently no established surgical guidelines or treatment algorithms for this disorder [3,4,5].

Surgical interventions commonly employed to address PP include soft tissue lengthening using Z-plasty techniques, muscle tendon releases of the knee flexors, knee capsulotomies, Ilizarov fixator placement across the knee joint, femoral extension osteotomy, neurolysis, and sciatic nerve lengthening. Despite these procedures, specific complications such as pin tract infections or paralysis of the sciatic nerve can arise. Additionally, there is a risk of recurrence, resulting in a relapse of the inability to ambulate, decreased range of motion in the knee joint, and recurrence of the equinus deformity [6].

In this case report, we present the unique instance of a one-year-old girl diagnosed with PPS. We discuss the challenges associated with the management and treatment of this rare syndrome and highlight the surgical interventions employed to improve her mobility and overall quality of life.

## 2. Case Demonstration

The patient was born with syndromic popliteal pterygium on the left side, causing an 80° flexion contracture of the knee and an 85° equinovarus deformity. This malformation was accompanied by other deformities, including syndactyly of the left foot, a misplaced bladder, urethro-vaginal fistula, and recto-vaginal prolapse. Initially, the focus of treatment was on addressing these complex symptoms. The parents had already consulted several pediatric orthopedic centers regarding the left leg. Prior to our treatment, an incisional biopsy along the cord was performed. During the surgery, there was an intraoperative suspicion that the sciatic nerve was running within the cord, causing tension on the popliteal skin web. Consequently, the operation was stopped, and amputation was suggested as an alternative.

**Figure 1:**
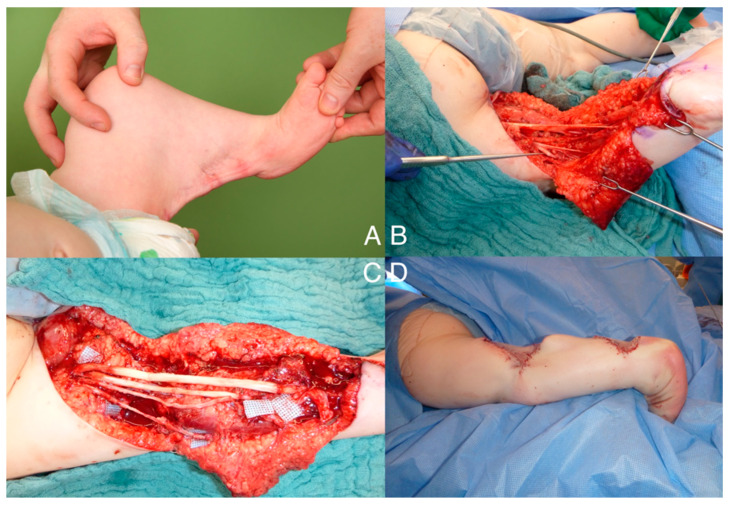
Demonstration of the popliteal pterygium. (**A**): fibrous cord resulting in a 85° flexion contracture of the knee. (**B**): after incision, sciatic nerve and popliteal vessels were found to be shortened due to the fibrous cord. (**C**): Interposition grafts of saphenous vein for popliteal artery and sural nerve grafts as well as allografts for tibial and peroneal nerve. (**D**): Closing of the skin could be achieved with Z-plasty, demonstrating a straight leg.

Subsequently, the parents brought the patient to our attention. We conducted a comprehensive interdisciplinary counseling board, explaining the different surgical options to the parents. From a microsurgical perspective, in addition to the planned orthopedic muscle and tendon lengthening procedures, as well as capsulotomies, we offered the interdisciplinary option of lengthening the shortened nerves and blood vessels. The parents were explicitly informed that nerve lengthening would initially result in complete denervation of the leg from the knee joint downward until reinnervation occurred. In terms of nerve reconstruction, it was anticipated that there would be insufficient autologous donor material available. Therefore, we extensively discussed the (at this time point in Germany) off-label use of Avance^®^ (Axogen, Alachua, FL, USA) nerve graft with the parents. The use of nerve conduits (e.g., chitosan conduits) and dermal substitute materials was also discussed. As this was the only treatment option compared to leg amputation, the parents expressly consented to all operative procedures (Figure 1).

At the age of 12 months, the patient underwent a surgical procedure involving soft tissue and nerve and vessel treatments. The surgery was performed under intubation anesthesia with the patient in a prone position. Before sterile cleansing and draping, an ultrasound examination confirmed the position of the popliteal vessels. The suspicion of the sciatic nerve running within the popliteal skin web could not be confirmed. Sterile cleansing and draping were carried out, and meticulous planning of the incision and extensive multiple Z-plasty were initiated. Soft tissue dissection was performed, revealing a thick fascia-like layer. The lesser saphenous vein and sural nerve were identified and dissected, with the latter being neurolyzed and divided for later grafting. The nerves in the lower leg were addressed, including the superficial peroneal nerve and deep peroneal nerve, which were dissected, neurolyzed, and preserved. The tibial nerve was also neurolyzed, and its motor branches were identified, dissected, marked, and preserved. Due to significant shortening, the nerve branches were transected and later lengthened using autologous material and additional nerve allograft. Tenolysis and Z-lengthening were performed on the hamstrings. The dorsal knee joint capsule was incised to enable full knee joint extension. Proximal lengthening of the gastrocnemius and soleus muscles and of the popliteus muscle had to be performed. Z-lengthening of the Achilles tendon and a capsulotomy of the ankle and subtalar joint were performed to correct the equinus deformity. The popliteal vessels were lengthened using lesser saphenous vein interpositions, and nerve reconstruction was carried out. The autologous ipsilateral sural nerve grafts were used to reconstruct the more important tibial nerve, the allograft was used to reconstruct the common peroneal nerve. Finally, wound closure was performed with the Z-plasty flaps and a plaster splint was applied. The surgery achieved a straight knee joint positioning, an 85° ankle joint position, and stable foot perfusion, while maintaining good capillary refill time. Perfusion was monitored hourly for 5 days according to the inhouse monitoring protocol used for free flap surgery. Due to the young age, there was no anticoagulant applied, but sufficient fluid management was performed.

In the postoperative period, the patient showed wound healing complications, which could be treated without additional surgery.

During the following years, interdisciplinary care with the technical orthopedic department was provided to the patient. To prevent scar contractures, to support the midfoot deformity, and to balance the leg length difference, individual orthoses were provided about twice a year.

**Figure 2:**
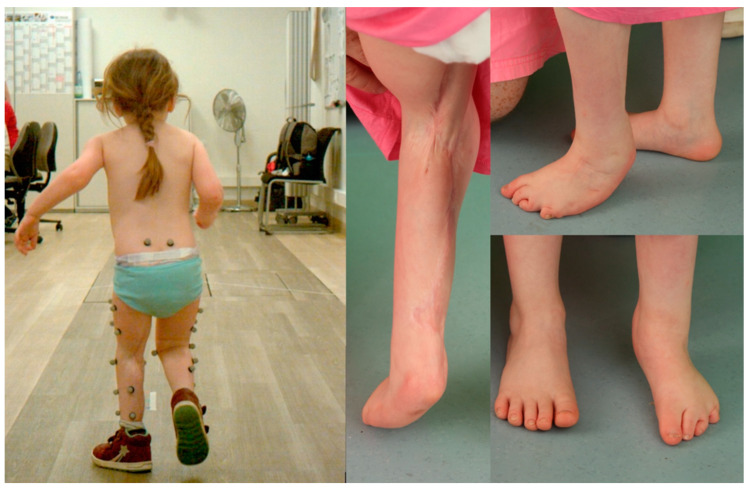
Walking ability during 3D motion analysis. A midfoot break and pes equinus is present three years after surgery as well as a difference of 2 cm in leg length.

At three-year follow-up, sensibility was reported in the entire limb. Tickling of the foot was immediately recognized by the patient. A Semmes–Weinstein (SWM) monofilament test was attempted as the compliance of the child in analyzing sensory discrimination was difficult to obtain. Hereby, the SWM test demonstrated clear recognition of FIN 6.45 (<180 g), but further clear sensory discrimination could not be achieved. In the clinical examination, there was a leg length discrepancy favoring the non-operated side of 2 cm. The hip joint range of motion was nearly normal with a slight hip flexion contracture of 5°. The patient showed an active knee range of motion of knee extension/flexion 0–20–90°. There was a fixed equinovarus deformity with 20° plantarflexion at the ankle joint. The patient is able to walk and run independently (Figure 2).

A 3D gait analysis could be performed with consistent data despite the young age of 4 years of the patient. The 3D gait analysis revealed—despite the ability to walk and run independently and the good active joint motions—several gait abnormalities. During the stance phase, there was an increased anterior pelvic tilt and pelvic internal rotation on the healthy side as a compensation mechanism to achieve forward motion. The knee of the involved limb maintained knee flexion of about 30° with only slight movement of 5° during the complete stance and swing phase, although active knee motion was possible. The equinovarus deformity with 20° of ankle flexion was compensated by the development of a slight midfoot break positioning.

Overall, the progress after surgery is highly satisfactory. She visits the Kindergarten and shows normal, age-related development. The patient is able to walk and run independently and has foot sensibility. While a minimal knee flexion contracture and equinovarus persisted, they do not currently cause functional limitations. Due to a midfoot break to overcome the ankle joint limitations, the foot will require a combination of bony and soft tissue foot correction as secondary surgery later on.

The 3D gait analysis provided valuable information on the patient’s gait abnormalities and guided the planning of further corrective steps. It highlighted the need for targeted interventions to address specific musculoskeletal issues, including the limited range of motion in the involved knee during gait, to improve their gait pattern and overall functional outcomes (Supplementary Video S1).

## 3. Discussion

Popliteal pterygium syndrome presents a complex set of musculoskeletal abnormalities that requires a tailored approach to treatment. In this discussion, we analyze the advantages and disadvantages of various treatment options for PP, including soft tissue reconstructions, muscle tendon releases, capsulotomies, Ilizarov fixator placement, femoral extension osteotomy, neurolysis and nerve lengthening, and the use of dermal substitute materials.

Soft tissue reconstructions aim to improve mobility and address the skin web, resulting in increased range of motion. The advantages of this approach include improved joint mobility and potential functional benefits. However, scarring and wound healing issues may occur as disadvantages, and deeper musculoskeletal problems may not be fully addressed. Additionally, there is a risk of recurrence/scar contracture, highlighting the importance of long-term follow-up [6,7].

Muscle tendon releases involve lengthening of tight muscles and tendons to improve joint mobility. The advantages of this procedure include potential long-term benefits and improved joint function. However, it requires specialized expertise and carries a risk of damage to surrounding structures. The extent of correction may also be limited, depending on the severity of the deformities [5,6].

Capsulotomies focus on releasing tight joint capsules to improve joint mobility and reduce contractures. The advantages of this procedure include improved joint mobility and the reduction in contractures. However, it may not address all components of PP, and there is a risk of joint instability or recurrence [6].

The placement of an Ilizarov fixator is another treatment option that allows for gradual correction of deformities and improvements in joint alignment. The advantages of this external fixator device include its ability to achieve progressive correction over time. However, it requires a longer treatment duration, regular adjustments, and close monitoring, and only less severe deformities compared to our case can be extended properly. Complications such as pin tract infections and discomfort may also arise. Grotesque malpositions of the femur and tibia as well as the corresponding joint surfaces in the knee joint can be the result [7].

Femoral extension osteotomy is a surgical procedure that corrects knee flexion deformities while maintaining the knee joint alignment. The advantages of this approach include its ability to correct the full amount of flexion contractures without compromising muscular function. However, the amount of correction angle is also limited and 80° of knee flexion contracture cannot be completely addressed [8].

Neurolysis and nerve lengthening aim to release and lengthen affected nerves, thereby restoring sensory and motor function. The advantages of this procedure include the reconstruction of nerve function and potential functional benefits. In particular, the protective sensation of the foot is highly important for secure ambulation. The parents of our patient described good sensation in the foot, which was in contrast to the monofilament test, although this was highly impaired due to the patient’s age. In the current literature, there are only four reports worldwide where the nerve was explicitly addressed, with two studies describing an expansion of neural tissue [9,10] and the other two using nerve grafts [11,12]. Detailed sensory outcomes after these procedures were not reported.

The use of dermal substitute materials provides additional support, promotes wound healing, and improves cosmesis. The advantages of these materials include their potential to enhance wound healing outcomes and improve the esthetic appearance. In our case, we were able to close the skin primarily using the Z-plasties, but in the case of a remaining defect, such dermal skin substitutes would have been used.

The best functional result of PP correction is achieved with a combination of all procedures to address all parts of the shortened soft tissue structures. Release must be performed from the sciatic tuber down to the calcaneus to address the whole cord, especially in severe forms with >40° flexion contracture. Microsurgical lengthening of vessels and nerves seems inevitable when performing single-stage straightening.

We found a difference in leg length of 2 cm in the 4-year-old child three years after the operation. This indicates that femoral shortening osteotomies performed to decrease flexion could result in further shortening of the leg and even more impaired gait behavior.

Considering the timing of the operation, an early age should be preferred as the fibrous cord is softer and joint contraction is still addressable. As the risk of anesthesia decreases significantly after the first year after birth, we would consider this the best age for the procedure.

It is crucial to consider individual factors such as the patient’s specific condition, age, and overall health when selecting the appropriate treatment for PP. A multidisciplinary approach involving pediatric orthopedic surgeons, plastic surgeons, and other specialists is often necessary to provide comprehensive care and optimize treatment outcomes.

## Data Availability

The data presented in this study are available on request from the corresponding author.

## Supplementary Materials

The following supporting information can be downloaded at: https://www.mdpi.com/article/10.3390/jpm14111097/s1, Video S1: Gait analysis after microsurgical reconstruction.
